# The Role of Microfluidics for Organ on Chip Simulations

**DOI:** 10.3390/bioengineering4020039

**Published:** 2017-05-04

**Authors:** Aziz Ur Rehman Aziz, Chunyang Geng, Mengjie Fu, Xiaohui Yu, Kairong Qin, Bo Liu

**Affiliations:** 1 Department of Biomedical Engineering, Dalian University of Technology, Dalian 116024, Liaoning Province, China; azizjatoi@hotmail.com (A.U.R.A.); 1352257919@mail.dlut.edu.cn (C.G.); krqin@dlut.edu.cn (K.Q.); 2 Dalian Institute of Maternal and Child Health Care. Dalian 116024, Liaoning Province, China; fmjnewlife@126.com (M.F.); yuxiaohui369@163.com (X.Y.)

**Keywords:** Microfluidics, 3D biopringting, Bioengineering, Biosensors, Microbiome

## Abstract

A multichannel three-dimensional chip of a microfluidic cell culture which enables the simulation of organs is called an “organ on a chip” (OC). With the integration of many other technologies, OCs have been mimicking organs, substituting animal models, and diminishing the time and cost of experiments which is better than the preceding conventional in vitro models, which make them imperative tools for finding functional properties, pathological states, and developmental studies of organs. In this review, recent progress regarding microfluidic devices and their applications in cell cultures is discussed to explain the advantages and limitations of these systems. Microfluidics is not a solution but only an approach to create a controlled environment, however, other supporting technologies are needed, depending upon what is intended to be achieved. Microfluidic platforms can be integrated with additional technologies to enhance the organ on chip simulations. Besides, new directions and areas are mentioned for interested researchers in this field, and future challenges regarding the simulation of OCs are also discussed, which will make microfluidics more accurate and beneficial for biological applications.

## 1. Introduction

Microfluidics, a multidisciplinary developing field, is related to small volumes, sizes, energy consumptions, and domains which are used to run, intermingle, discretize, or process fluids [1,2] for better analysis of single cells to organs on fully automated chips [3]. Two dimensional (2D) cell culture systems are not reliable for predicting many cellular functions like drug activities, and for controlling the precise physical and chemical microenvironments. These systems do not fulfill the main requirements of cellular microenvironments in vivo [4]. So, they are replaced by three dimensional (3D) systems which better represent the interactions of factors and the complexity of tissues. However, organoids, formed in 3D cell cultures, have different shapes and sizes and it is hard to maintain the positions of their cells for analysis. Functional analysis, i.e., trans-cellular transport, secretion, and absorption, and biochemical and genetic analysis of cultured cells, are also difficult in 3D systems. In many systems, multi-scale architectures and the tissue-tissue-interfaces, such as vascular endothelium and the media layers, are missing. There is a lack of exposure to mechanical cues like tension, compression, and flow shear stress (FSS) for cells, which are very important for organ development and functions for both disease and health states [5]. Microfluidic systems offer opportunities to overcome these limitations.

An organ is a complex unit of different tissues, and these tissues are composed of various types of cells with diverse functions. Organs of the human body can be simulated by microfluidic devices. These microfluidic devices usually contain cell culture chambers lined with living cells. For the simplest system, there is only one single cell lining which represents corresponding tissue functions [6]. However, for complex systems, different types of cells related to various tissues are cultured. These cells are interconnected with porous membranes and are lined on opposite sides for proper organ simulation [7]. Microfluidic devices can be used to mimic the organ functionality by multi-cellular architecture, interfacing the tissues and physiochemical microenvironments along with perfusion of the body. Fluidically associated cells of different organs on a chip, mimicking the physiological connections between organs with integrating FSS, mechanical compression, and cyclic strain or other physical forces, can be used for analyzing drug distribution and organ specific responses [6]. Electrochemical monitoring, selective cell attachment patterns, sensing systems for electrochemical transductions, and enzymes and biomarkers in microfluidic devices can be achieved by the integration of some supporting technologies with these devices.

Although better results can be achieved by mimicking the in vivo microenvironment using microfluidic systems as compared to other traditional systems, for simulation from the cell to tissue level, more complex environmental conditions are required. These enhanced prerequisites make simulations more difficult. Similarly, for organ simulation, where two or more dissimilar tissues are associated with each other causing tissue-tissue-interactions and greater intervening factors, the conditions for simulation become stricter. When discussing the disease model of an organ, this complexity is further augmented. So, for organ simulation involvement of different parameter increases, microfluidics alone cannot control all these parameters precisely. Fortunately, it is not necessary to form a complete working organ, but a functional unit that can be synthesized, which summarizes the organ level functions. However, some other technologies are still needed to control these parameters.

## 2. Organ Simulation Is Better with Microfluidics 

The following characteristics and advantages of microfluidic systems make them more reliable and competent for better physiological analysis and for controlling system parameters more precisely than other systems. (1) The flexibility of microfluidics can provide physiological microenvironments using perfusion or 3D tissue like structures. In 3D cell culture, cells are embedded in natural or synthetic polymers. These natural or synthetic polymers may be collagen, matrigel, and hydrogel. In microfluidic channels, hydrogels are widely used for cell encapsulation [8]. Many models are formed by hydrogels, e.g., cells intersperse randomly in the extracellular matrix (ECM) or form organoids which are clusters of self-assembled cellular structures. These 3D models are useful for studying signal pathways, drug responses, and tissue functions [9,10,11,12]; (2) In chips, the fluid flow is vital because at the smaller scale, viscous forces are dominant. If the diameter of the channel is less than 1 mm, the flow is laminar and allows for the production of chemical and physical gradients. These gradients have been used in studying cardiac tissue formation, directional cell migration [13], nerve axon outgrowth [14], differentiation [15], graded metabolism [16], neurotoxin responses [17], cell-cell junction integrity, and sub-cellular structures [18]. Microfluidic systems provide vasculature on chips for studying drug screening [19]. The FSS can be controlled, irrespective of gradients, by changing the channel dimensions and/or flow rates, by using a nonporous membrane which separates cells from the flow path or restricts the cell passage by micro-engineered posts; (3) Cell survival and functions can be enhanced by fluid mechanical computational models. These models optimize the micro-channel geometry which increases nutrients and oxygen delivery; (4) The complex mechanical microenvironment of organs can be summarized in vitro by chips; (5) Flexible side chambers and cyclic suctions can be used to create cyclic mechanical strains [7,20,21,22]. Cells can be exposed to FSS and cyclic mechanical deformations like the living cells which are involved in the processes of cardiovascular cycling, breathing, and peristalsis [7,20,21,23]. Similarly, enhanced pressure can be applied to compress the tissues which normally respond to compression [24]; (6) In the micro-channel, different cell types can be placed in different patterns or in direct concurrence on the same planer substrate by many methods. For example, laminar streams can be used to plate ECM proteins or cells [25]. Complex micro-channel path designs can be applied to make contact with the adhesive substrates. Micro-posts can be situated between adjacent cells [26] or ECM can be printed in different positions in micro-channels [23,27]; (7) Using molding techniques, substrates can be fashioned into organ-like forms, e.g., the villous shape of intestine [28]. Chip designs include implanted cells in 3D ECM gels [29,30,31,32,33] and multi-cellular constructs using tissue engineering (TE) [23,33]. The integration of porous substrates for separating two micro-channels helps to analyze the absorption, secretion, transcellular transport, and tissue-barrier functions [7,20,21,22,34,35,36]. Tissue-tissue-interfaces can be produced by culturing different cells on opposite sides of the substrate. These interfaces can mimic the interactions of parenchyma and vascular endothelium tissues, i.e., identified by almost all organs [7,20,36].

Organ on chip (OC) is beneficial in investigating organ physiological and disease models. It is suitable for analyzing tissue architecture and perfusion dependent biological mechanisms. OC can be applied to different diseases or drug studies as an alternative to other cell cultures and animal models. Large numbers of samples are easier to handle on a chip and it also increases the significance of results. Due to its ability to control fluid-flow, cell survival and differentiation can be increased, e.g., lung has been cultured for one month on a chip [20]. In the future, it can also help to study chronic pathophysiological responses, computational modeling for fluid-dynamical interactions with metabolites, and cell and gas interactions with circulating cells, e.g., blood, tumor, immune cells, and bacteria [21].

## 3. Limitations of Organ Simulation on Microfluidics

Microfluidics is a field of engineering, while in organ simulation it is being used mainly by the biomedical community. Thus, researchers will face some integrating problems of the two separated fields [37,38]. Currently, co-culture systems within microfluidic models are widely used for OC. For example, Huh et al. fabricated a lung model on a chip with epithelial and endothelial cells on a porous membrane [20]. Microfluidics is very beneficial in simulating different organs, but only microfluidic technology is not sufficient for simulation of all organs. For example, for the simulation of the heart and nerves, it is interlinked with electrodes to produce an electrochemical environment for their proper simulation [38,39]. Although a lot of work has been done and published in this new field, organ simulation with microfluidics is still in its infancy, and further development is required to overcome its limitations. Many physical and chemical factors become dominant at the micro-scale, and can influence the results. For example, the surface area and its roughness affect not only the capillary forces but also the flow rate of the micro-fluid, and it is hard to neglect the chemical interactions. The chemical nature of the construction material affects the reaction processes. These unnecessary interactions and interventions influence the accuracy of the results. These types of physical and chemical factors make the microfluidic organ simulation more difficult and it requires the coupling of supporting technologies. During micro-fabrication, it is necessary to keep high accuracy and precision according to the principles of engineering [40]. In addition, the chemicals and chips used in these processes are disposable and re-sterilization is very difficult if reuse is necessary. OC is not reliable for certain areas, such as adaptive immune responses, responses to endocrine systems, nervous systems, skeletal systems, and chronic diseases [6]. They are mostly good for studying diseases of short time frames depending upon the cell positions in the micro-architecture. However, researchers still need to improve the efficiency of organ simulation by completely understanding the micro-environmental factors, which regulate the cell differentiation, development, disease progression, healing and regeneration, immune cell contribution to toxicity, infection, inflammation, and multi-organ failure. Combining induced pluripotent stem (iPS) cells or patient-specific primary cells along with gene editing technologies and personalized models of disease and health needs to be developed. Up to now, the complexities of organ functions and other requirements for its simulation on chip have not allowed researchers to replace human testing [6,40,41]. Selective cell attachment patterns, detection systems for electrochemical transductions, fluorescence, enzymes, biomarkers, genomics, proteomics, lipidomics, electrochemical monitoring, and viable tissue production cannot be achieved by microfluidic systems, so the integration of other supporting technologies are needed for supporting and enhancing the simulation of OCs.

## 4. Microfluidics Devices Accompanied with Supporting Technologies for Better Simulation

Organ simulation is more complex because of the enhanced environmental conditions and requirements. For organ simulation, microfluidic platforms can be integrated with additional supporting technologies to enhance OC simulations. Below are some technologies which can be integrated with microfluidic systems to mimic the microenvironment and simulate organs more precisely.

### 4.1. 3D Bioprinting

3D bioprinting technologies are involved in creating cell patterns where the functions and viabilities of cells are preserved in printed constructs [42] by layering different biomaterials and cellular compositions [43]. Bioprinting can produce organ and tissue like structures which can be used in research [44]. Bioprinting creates complex design channels and draws or digs connectors by fabrication. Microfluidic devices with automated 3D fabrication are produced with 3D technology [45]. Therefore, 3D printing of an OC is possible. With the convergence of bioprinting and microfluidic OCs, complex artificial tissues are formed which have human body like micro-architectures for chemical and mechanical stimuli [46]. For example, for drug testing, liver on a chip (LoC) was developed for the long term culture of 3D hepG2/C3A spheroids using a bioreactor design. This engineered bioreactor could interface with a bioprinter for fabricating 3D hepatic constructs, which remained functional for a 30 day cultural period [47]. Recently, by bioprinting hepatic spheroids, a device conducive to hepatotoxicity analysis was developed [48]. This microfabrication allows for better control over the microenvironment for stabilizing the liver for weeks [49]. Now various designs of the bioprinted LoC have been demonstrated for the different functions of the liver [50]. The need for blood vessels makes cardiovascular organoid simulation more complex. Now, 3D bioprinting is being used to fabricate endothelialized myocardium for better analysis and disease modeling [51,52,53]. The 3D bioprinted microfluidic systems with complex biological culture systems improve the proximal tubule functionality and epithelial morphology [54]. Johnson et al. demonstrated the extrusion-based 3D cell printing model, used for nervous system modeling [55]. The future of this technology is 4D bioprinting, which may be helpful in exploring the functional biological constructs [56].

### 4.2. Biosensors

Combining biosensors with microfluidic devices helps to improve the performance of sensing systems by improving the transport of analytes. Reduced volumes and distances in microfluidics make the biorecognition of elements easier. These results can further be improved by specially designed channels. There are different types of biosensors used for different purposes. Some microfluidic biosensors are based on electrochemical transductions. These biosensors can be used in heart and nerve microfluidic devices [57,58]. Microchambers of polydimethylsiloxane (PDMS) with electrodes and sensors can monitor cardiomyocytes optically and electrochemically [59]. Electrical field application is studied in contractile cells [23] and wound healing [60]. This technology has been mostly applied to primary/established cell lines up to now, but it can be used for any cultureable cell line including insects and plant cells for the identification of toxic pesticides/defoliants to humans. An organo-typic model of nerve tissues mimicking nerve compound action and nerve fiber density may be more useful in clinical outcomes. However, physiology, neurological architecture, and the surrounding ECM are very hard to mimic. A study describes the electrophysiological-recording of intra and extra cellular recordings with a micro-engineered sensory neural fiber tract [61].

Enzyme-based detection has also been improved in microfluidics. Different enzymes have been detected by using these types of biosensors [62]. Electrochemical biosensors can be applied for analyzing affinity-based biorecognition events. Electrochemical signals can also be recorded from binding events. Amperometric detection is common, but electrochemical impedance spectroscopy can also be applied [63,64]. Similarly, other biosensors are fluorescence based and non-fluorescence based biosensors, micromechanical transduction based, thermal, magnetic, magnetoresistive sensors, etc., which can be linked with microfluidics and their linkage can provide crucial information which microfluidics cannot provide alone [63]. Bioreactors linked with electrochemical sensors provide the functional status of cells or specific organelles, and real-time monitoring of oxygen uptake [65,66]. A microfluidic thermal biosensor is designed to detect L-glutamate which can be helpful in recognizing the dynamics of the neurotransmitter [67]. The 3D microfluidic devices are also improved by culturing techniques to detect the biomarkers of drug interactions and drug induced injury in the kidney. A biosensing platform for protein detection was proposed, which can be used for the detection of biomarkers [68]. Sometimes traditional analyses like immunoflurescence microscopy, microplate immunoassay, and immunoblot are also required. The incorporation of a fluorescence-nanoparticle immunoagglutination/immunocapture assay into OC allows dual mode monitoring for drug toxicity [69,70]. Recently, a smart phone based, fluorescence-microscope was fabricated for monitoring the device attached with OC, and this dual mode monitoring permits both internal and external monitoring of OC [70].

### 4.3. Multi-Electrode Array (MEA)

**Figure 1:**
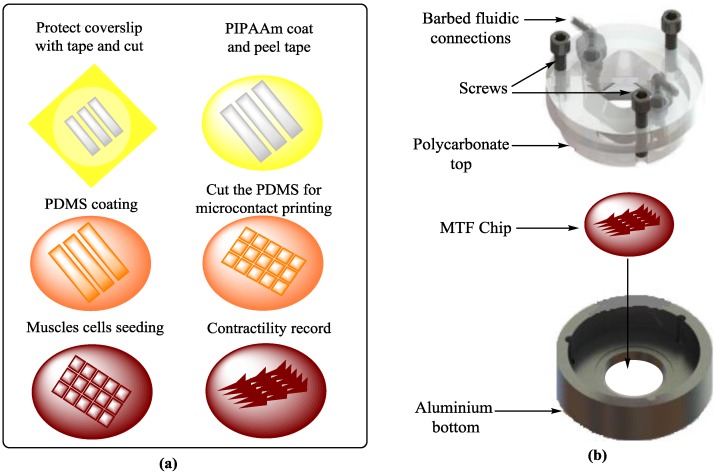
Heart on a chip [23]: (**a**) Scheme of the fabrication process; (**b**) Assembly of the microfluidic device, consisting of an aluminum bottom, a recess for holding the chip, polycarbonate top held by three screws, and barbed fittings with fluidic tubes for fluidic input/output.

Electrochemical signals are produced in cardiomyocytes due to their alterations in different ionic concentrations at cellular levels, in which the calcium ion concentration and membrane depolarization are responsible for the contractile force and cell shortening due to the initiation of actin-myosin motors. Similarly, nerves also show electrophysiological behaviors. These electrophysiological and contractility responses have caused many problems for mimicking the replication of nerve and cardiac tissue environments in vitro. The simulations of heart and nerve are interlinked with electrodes to produce an electrochemical environment for proper simulation. Electrophysiological techniques are applied by a multi-electrode array (MEA) for detecting environmental toxins [71,72], therapeutic testing, and disease modeling [73,74] in nervous systems. The integration of muscular thin films (MTFs) into microfluidic devices [23] helps in exploring the fluid-flow, tissue-tissue-interactions, and electrical and mechanical cues involved in the development of heart diseases (Figure 1). The contractility measurements of engineered 3D cardiac tissue constructs are made either isometrically or auxotonically against the applied force. The I-wire platform is used to control the applied force which creates the electrical and mechanical characterization of these tissues. Therefore, it is valuable for studying cardiac diseases, drug development, and screening [75]. Recently, a microfluidic device [76] including a hanging-posts-array and a pneumatic-actuation-system to confine cell laden gels and homogeneous uniaxial cyclic-strains to cell constructs during culture, respectively, was developed for generating mature microengineered cardiac tissues. This model presents a step forward in this field and provides a 3D functional cardiac model [76]. A microfluidic chip containing thousands of microelectrodes was designed and tested for bacterial sterilization using a pulsed electric field. The sterilization of bacteria as a function of the strength of this electric field, width and pulse number, treatment buffer, bacterial growth, and bacterial enrichment with positive dielectrophoresis were experimentally analyzed on the chip. It was observed that 100 V was enough for good sterilization. The configuration of the microelectrode arrays influenced the bacterial sterilization. Firstly, the bacteria were concentrated in the high electric field region by dielectrophoresis and then the enriched bacteria were killed by the pulsed electric field through microelectrode arrays [77].

### 4.4. Tissue Engineering (TE)

**Figure 2:**
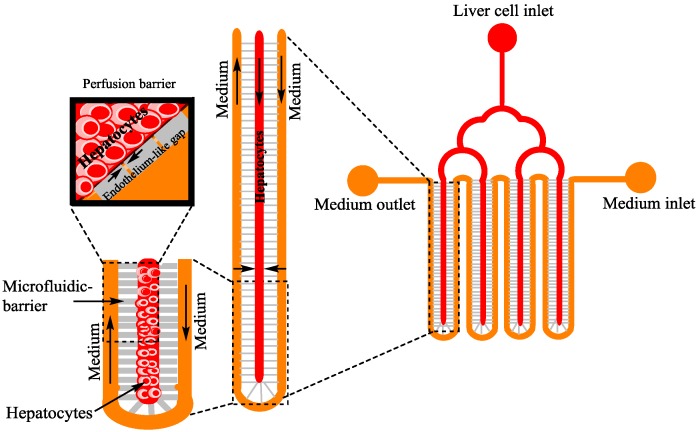
Liver on a chip [79] with cell culture: Red colour represents the hepatocytes, gray colour represents the endothelium like gaps, and yellow colour represents the medium. Channels are separated with microfabricated barriers for the separation of hepatocytes from fluid.

Electrochemical tissue engineering (TE) is useful in improving or replacing biological functions. It involves new viable tissue production for medical purposes. Cell-cell interactions, i.e., homotypic and/or heterotypic interactions, maintain tissue functions and structures, and many cells respond to these interactions. For example, LoC mimics heterotypic interactions via separating hepatocytes, cultured in low shear stress and diffusion prevailing microenvironments, at a high homotypic cell-density. A silicon micro-machine comb [78] is used to control the spacing among the cell populations, and the patterns of stamped substrate interactions between the fibroblast and hepatocytes (Figure 2).

**Figure 3:**
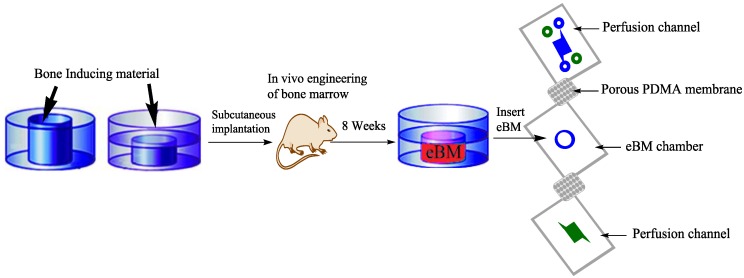
Bone on a chip [83]: Polydimethylsiloxane (PDMS) device with cylindrical cavity filled with induced bone material (open at either ends or closed at one end) is to form engineered bone marrow (eBM) in vivo for 8 weeks and then this eBM is cultured in a microfluidic device. The green and blue channels are medium perfusion channels for maintaining the eBM in the central chamber.

Recently, a liver lobule-like structure was constructed on a chip by a microfluidics-based bioengineered strategy, which was used for exploring liver tissue engineering, pathological studies, and drug induced toxicity [80]. In 3D systems, the integration of synthetic polymer gels into microfluidic channels gives greater complexity to the tissue microenvironment on a chip. Uniform cell aggregated co-cultures of hepatocytes and fibroblasts are encapsulated in 3D hydrogels for the production of hepatic micro-tissues [81]. These micro-tissues can be individually harvested over time in the device and exposed to drugs and fluids [81,82]. Yu-suke Torisawa has introduced bone on a chip [83], which contains complex bone marrow by uniting microsystems with TE. TE has evolved from biomaterials development. It features the combination of cells, material methods, and engineering physiochemical and biochemical factors to replace/improve tissues [84]. A PDMS device with a central cylindrical cavity (open at both sides) was designed and implanted on the back of mice after filling the cavity with bone inducing materials [83] (Figure 3). A bone marrow containing bone resulted after eight weeks, which was confirmed by its identical morphology comparison to natural bone. Another microfluidic device was used for culturing engineered bone marrow (eBM) and that eBM was removed surgically and placed in a microfluidic device [83]. The eBM’s cells kept their viability after 4 days of culture, and the results showed similarity to the mouse femur BM, but not to blood cells. This type of bone simulation with microfluidics was supportive in learning about drug discovery research, transplantation of BM, and hematopoietic stem cell diseases. The combination of mechanical forcing regiments and fluid-flow, similar in vivo, could improve tissue and organ functions. When adipocyte derived cells and bone marrow on a chip were exposed to dynamic hydraulic compression, enhanced bone differentiation was measured [24]. Recently, Torisawa et al. used that model to demonstrate the bone marrow responses in vitro and blood cell production to radiation countermeasure drugs [85]. The association of TE and microfluidics also improved the functional analysis of other organs such as lung on a chip [86] and heart on a chip [87]. Conventional micro-electromechanical systems technologies, in the field of TE, have also been used for fabrication on silicon wafers. These technologies are expensive and less developed for biological processes. The digital micro-mirroring microfabrication system incorporates a dynamic mask-less fabrication technique and utilizes its digital micro-mirrors for fabricating the biological devices. This microfabrication system provides the fabrication of biological microfluidics, designed to mimic in vivo conditions [88].

### 4.5. Omics and Microbiome

**Figure 4:**
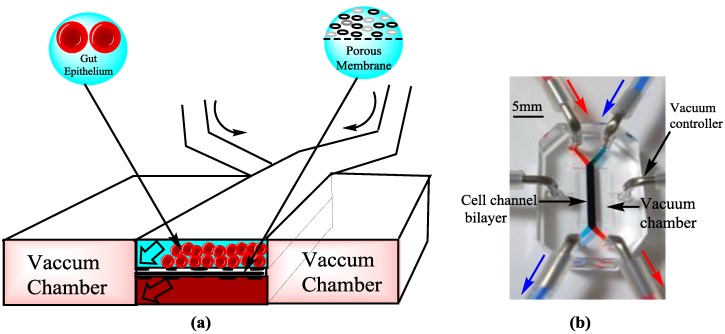
Gut on a chip [21]: (**a**) Gut on a chip device showing an extra cellular matrix (ECM) coated flexible porous membrane with gut epithelial cells through the middle of the central microchannel and vacuum chambers on both sides; (**b**) An image of the gut on a chip device. Syringe pumps are used to perfuse the blue and red dyes (directions indicated by arrows) to visualize these channels.

Omics technology is related to the collective characterization and quantification of biological molecules, which are translated into the structures, functions, and dynamics of organisms. Bioinformatics or other database related work integrations with microfluidic systems are also important achievements. Coupling microfluidics and micro-wells can be used to compare and profile the gene expression of thousands of single cells from different or similar experimental conditions [89]. Microfluidic devices are designed to separate neutrophils from blood for genomics and proteomics [90]. Similarly, microfluidic devices based on electro-spray ionization increase lipid separation and the identification of individual molecular species is extended by multidimensional mass spectrometry for lipidomics [91]. Electrochemical microfluidic devices are further developed by co-culturing the living micro-biomes with micro-engineered intestinal villi (Figure 4). This protocol can provide a platform for host-microbiome ecosystems, found in other organs. It can be helpful in exploring the role of the human-microbiome in health and disease states [92,93]. Omics technology, gut on a chip, and bacterial engineering are being used to treat intestinal diseases [94]. Hence, Omics technology and the microbiome in gut on a chip system made it physically and functionally analogous to human intestine and could be used for studying the major unsolved issues of intestinal diseases, drug testing, and toxicities. This can also facilitate research in the field of antibiotics and bacterial infections [95].

## 5. Future Opportunities and Challenges

Problems in the field of simulations of OCs can be solved by the combination of other technologies and can be used for the improvement of human health. Significant work has been done in this field but there are still many opportunities and challenges. (1) Many early challenging applications of these systems include the analysis of cells and fluidic optics, organic synthesis, detectors, high throughput screening, microelectromechanical systems (MEMS), and extension into nanofluidics. (2) Mimicking of human organs and their physiological responses is being made more affordable so that biomimetic microfluidic systems should be efficient enough to replace animal testing in the future. A computer simulated functional human model of multi-organ toxicity (four organ system) was established for evaluating multi-organ toxicity. The results obtained from this model agree with the results from both human and animal models [96]. (3) Bubble formation has been a serious problem in the operation of microfluidic devices [97]. A new perfusion process needs to be developed for mimicking the cell environment, which will provide a new platform for testing, possibly for the production of closer in vivo mechanisms that affect absorption, elimination, distribution, and metabolism. (4) Pharmacokinetic models, by which the fate of drugs can be studied in organisms, can be improved [38,39]. The use of iPS cells is beneficial in designing diseased organs. Current protocols generally produce immature cells, e.g., hepatocytes, cardiomyocytes, and endothelial cells [98,99,100]. This problem is being solved by multiple approaches, e.g., the use of progenitors and through the identification of small molecules which can establish renewable sources of hepatocytes [101,102]. The culture of stem cells of a specific lineage, in a physical microenvironment, on a chip may also be valuable for differentiation, and finally these lines may lead to a human on a chip with all the organs from one patient [6]. (5) The fabrication of OCs needs to be improved. Some polymers such as polyurethane elastomers are found to be resistant to small hydrophic compound absorption [103], but a suitable material is still in demand in this field. ECM coating degradation or contraction may also be a problem. Avoiding microbial contamination, culturing healthy cell seeding, the maintenance of cell-cell interactions, and ECM-cell interactions for precise tissue structures and functions are still challenges. (6) With the increase in functionality and complexity, from the cell to organ level, high resolution imaging becomes difficult because it is difficult to visualize processes in complex living systems. It is required to combine these systems with microfluorimetry, fluorescence confocal microscopy, trans-epithelial/endothelial electrical resistance (TEER) measurements, and electrodes for better analytical assays. TEER measurements are indicators of the integrity of cellular-barriers, used for measuring the integrity of tight junction dynamics in endothelial and epithelial cell cultures [104]. In the future, micro-sensors, such as molecular reporters, may be linked to chips using microscopes and robotic systems for better imaging [6,40,41]. A universal blood substitute is needed. Scaling approaches should be improved for accurate fluid-flows, appropriate organ functional activity, tissue masses and volumes, and computational pharmacokinetics/pharmacodynamics models [3,105].

## 6. Conclusions

The complexities of organ functions and other requirements for their simulation on a chip will not allow researchers to replace human testing so easily. Although preliminary microfluidic platforms have helped researchers with OC simulations, the next generation of microfluidic platforms needs to be integrated with diverse technologies to enhance their capabilities. Some of these techniques could be 4D bioprinting, automated instruments, advanced TE methods, and sensors of functional parameters, e.g., flow, pressure, pH, temperature, glucose, lactate, oxygen, electrical conduction, and TEER, linked with microscopic-microfluorimetric imaging for monitoring system performance.
